# Supplementary material for: UV LED Curable Perfluoropolyether (PFPE)-Urethane Methacrylate Transparent Coatings for Photonic Applications: Synthesis and Characterization
Source: Polymers (Basel). 2023 Jul 8;15(14):2983. doi: 10.3390/polym15142983 (PMC10383132; doi:10.3390/polym15142983)
Supplement: Supplementary file 1 [file polymers-15-02983-s001.zip › polymers-2429156-SI.pdf]

polymers

MDPI

Supplementary materials

UV-LED curable perfluoropolyether (PFPE)-urethane methacrylate transparent coatings for photonic applications: synthesis and characterization

Christian Dreyer [1,2], Dana Luca Motoc [3,*], Mathias Koehler [2] and Leonid Goldenberg [1,2]

[1] Technical University of Applied Sciences Wildau, Fiber Composite Material Technologies, Hochshulring 1, 15745 Wildau, Germany
[2] Fraunhofer Institute for Applied Polymer research IAP, Research Division Polymeric Materials and Composites PYCO, Schmiedestr. 5, 15745 Wildau, Germany
[3] Transilvania University of Brașov, Faculty of Mechanical Engineering, Department of Automotive and Transport Engineering, 29 Eroilor Av., 500036 Brașov, Romania
*Author to whom correspondence should be addressed: danaluca@unitbv.ro

Table S1. Correlation matrix accounting conversion values - free side

	Exposure rate (mm/s)	Conversion PFPE-U(MA/A)	Conversion PFPE-U(MA/A)-DM(DV)S	Conversion PFPE-U(MA/A)-TM(V)S	n PFPE-U(MA/A)	n PFPE-U(MA/A)-DM(DV)S	n PFPE-U(MA/A)-TM(V)S
Exposure rate (mm/s)	1.000						
Conversion PFPE-U(MA/A)	-0.821	1.000					
Conversion PFPE-U(MA/A)-DM(DV)S	-0.940	0.880	1.000				
Conversion PFPE-U(MA/A)-TM(V)S	-0.977	0.888	0.982	1.000			
n PFPE-U(MA/A)	-0.997	0.803	0.953	0.977	1.000		
n PFPE-U(MA/A)-DM(DV)S	-0.889	0.940	0.924	0.910	0.888	1.000	
n PFPE-U(MA/A)-TM(V)S	-0.977	0.788	0.861	0.920	0.964	0.873	1.000

Table S2. Correlation matrix accounting conversion values – glass side

	Exposure rate (mm/s)	Conversion PFPE-U(MA/A)	Conversion PFPE-U(MA/A)-DM(DV)S	Conversion PFPE-U(MA/A)-TM(V)S	n PFPE-U(MA/A)	n PFPE-U(MA/A)-DM(DV)S	n PFPE-U(MA/A)-TM(V)S
Exposure rate (mm/s)	1.000						
Conversion PFPE-U(MA/A)	-0.978	1.000					
Conversion PFPE-U(MA/A)-DM(DV)S	-0.983	0.964	1.000				
Conversion PFPE-U(MA/A)-TM(V)S	-0.954	0.926	0.887	1.000			
n PFPE-U(MA/A)	-0.997	0.962	0.989	0.941	1.000		
n PFPE-U(MA/A)-DM(DV)S	-0.889	0.924	0.945	0.730	0.888	1.000	
n PFPE-U(MA/A)-TM(V)S	-0.977	0.988	0.949	0.962	0.964	0.873	1.000

Table S3. Scattered light intensity variation function of distance from the coupling point.

Specimen	@633 nm	@1547 nm
PFPE-U(MA/A)	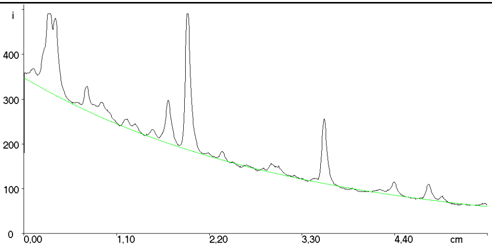	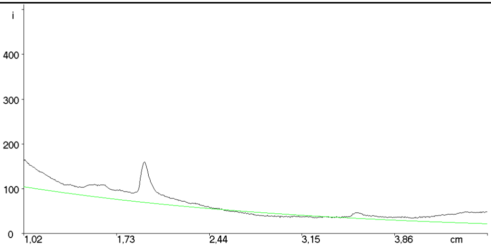
PFPE-U(MA/A)-DM(DV)S	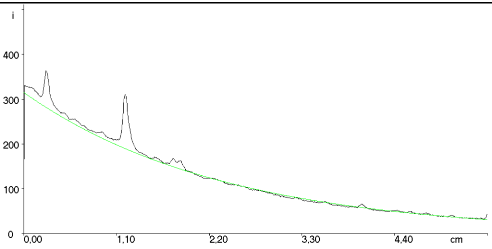	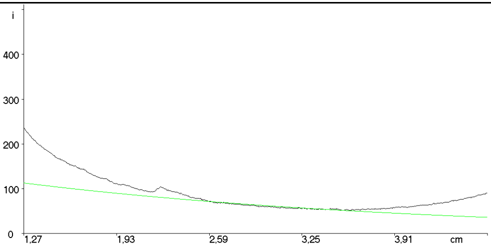
PFPE-U(MA/A)-TM(V)S	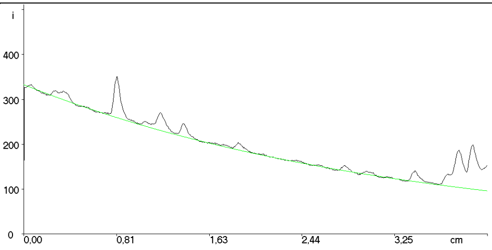	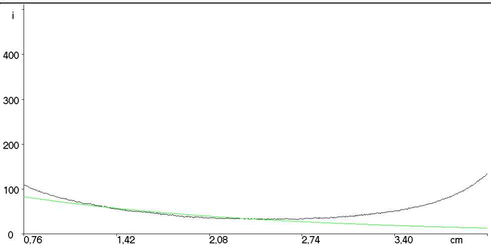
